# MYXOID NEUROTHEKEOMA: A RARE SOFT TISSUE TUMOR OF HAND IN A 5 MONTH OLD INFANT

**DOI:** 10.4103/0019-5154.48990

**Published:** 2009

**Authors:** Hussah Al-Buainain, Kamalesh Pal, Hossam El Shafie, Dilip K Mitra, Mohammed A Shawarby

**Affiliations:** *From the Division of Pediatric Surgery, Department of Surgery, College of Medicine, King Faisal University, King Fahad Hospital of University, Al Khobar, Saudi Arabia*; 1 *From the Division of Pediatric Surgery, Department of Pathology, College of Medicine, King Faisal University, King Fahad Hospital of University, Al Khobar, Saudi Arabia*

**Keywords:** *Infant*, *neurothekeoma*, *soft tissue tumor hand*

## Abstract

Myxoid Neurothekeoma is a rare benign nerve sheath tumor, commonly seen in young females. Patients usually present with a small nodule in different anatomical sites, commonly involving the face and the upper limb. We present the case of a five-month-old boy, who presented with a nodule on the left thumb. Punch biopsy and immunostaining confirmed the diagnosis of myxoid neurothekeoma. We believe this is the first reported case of myxoid neurothekeoma below 12 months of age.

## Introduction

Neurothekeoma is a rare benign soft tissue tumor with fairly distinctive histological features. It is commonly located on the upper extremities or the head and the neck.[1] Histologic variants include myxoid, cellular and mixed tumors. A recent immunohistochemical pattern enables a differentiation between myxoid neurothekeoma, melanocytic and nervous system tumors.

Complete excision is the mainstay of treatment for neurothekeoma. Recurrence is attributed to incomplete excision. We present the first case report of rare myxoid neurothekeoma in an infant of 5 months’ age and discuss the histopathological features distinguishing it.

## Case Report

**Figure 1:**
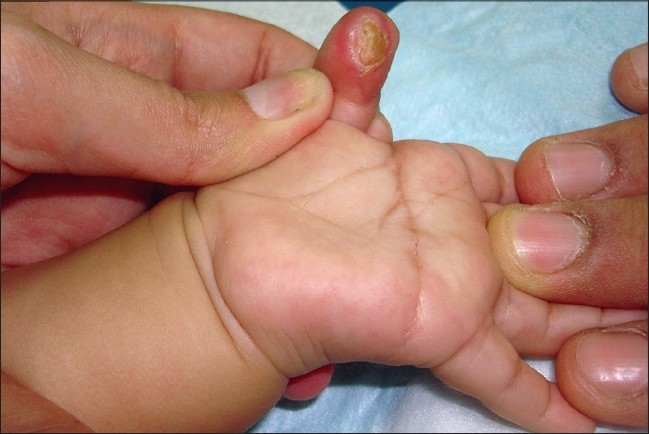
Soft tissue nodule measuring 5 × 5 mm^2^ on the ventral surface of thumb

**Figure 2:**
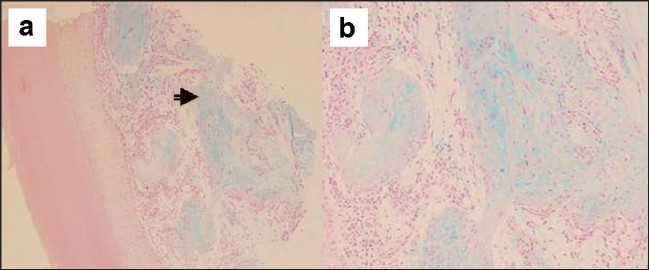
Well circumscribed lobular masses of spindle cells (arrow) in an abundant alcian blue positive mucoid (myxoid) stroma in dermis. × 100(a), × 200(b)

**Figure 3:**
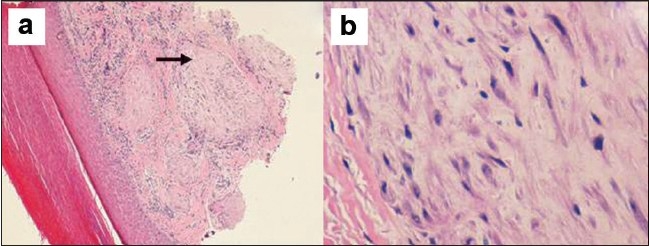
Hypocellular lobular mass of bland spindle cells in the dermis (H and E stain, ×100 (a), ×400 (b)

**Figure 4:**
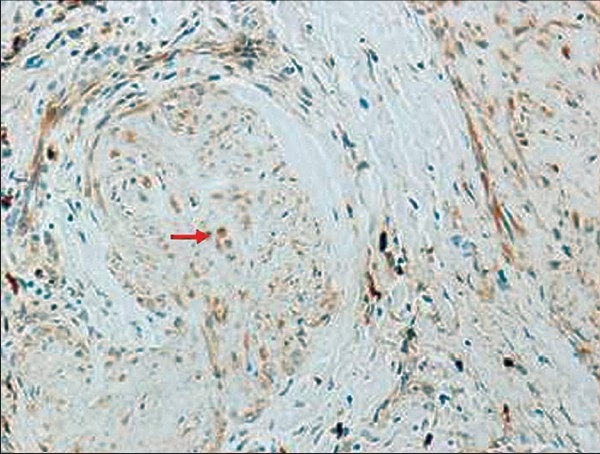
S-100 positive cells (arrow) within hypocellular lobular mass

A five-month-old boy was brought with left thumb swelling of three weeks’ duration. It was a 0.5 × 0.5cm^2^, pinkish red, firm, immobile, non tender, non fluctuant intradermal swelling, with intact overlying skin [Figure 1]. There was no history of trauma. Punch biopsy revealed fairly well circumscribed hypocellular lobular masses, with abundant spindle cells in the dermis, positive staining for S100, alcian blue and mucoid stroma [Figures 2–4].

## Discussion

Soft tissue tumors are uncommon in the pediatric age group and differ from adult incidences in different aspects, including frequency, anatomical site and prognosis. Differential diagnosis of the dermal nodule in infancy and young adulthood should include fibrous tumors, histiocytic tumors, lymphocytic tumors, melanocytic tumors and neural tumors.[2]

Neurothekeoma is a rare benign tumor of the nerve sheath, with a distinct histomorphological character. It was described initially by Hakin and Reed, in 1969, as Nerve sheath myxoma. The term Neurothekeoma was described by Gallager and Helwing, in the 1980s.[3] There is an overlapping feature with other neural tissue tumors such as schwannoma, nerve sheath myxoma and neurofibroma, leading to difficulties in diagnosis.

This disease is commonly present as small solitary nodules on the face and the upper limb; however, other anatomical sites have been reported, such as oral cavity, cauda equina, shoulder and neck.[4]

Based on histomorphological appearance and immunohistochemical findings, there are three variants of neurothekeoma–myxoid (classical or hypocellular), cellular and mixed type.[5]

The myxoid type is characterized by greater degree of myxomatous changes, less cellularity with well-circumscribed spindle cells in myxoid matrix and multinucleated giant cells; they, characteristically stain positively for S-100, collagen type IV and nerve growth factor receptor; and, are negative for epithelial membrane antigen or markers of histiocytic differentiation. In contrast, the cellular types of neurothekeoma are not encapsulated. The cells are epithelioid with eosinophilic cytoplasm and have rare mitoses; they do not stain S-100, collagen type IV, or nerve growth factor receptor but show reactivity with NK1C3 (CD57) and the panmonocyte marker Ki-M1p.[6] The mixed type of neurothekeoma shows areas of varied cellularity with focal myxoid regions.

Dual immunoreactivity for neuron specific enolase and S-100 protein in myxoid type support Schwann cell origin, while the absence of S100 protein and positive epithelial membrane antigen in the cellular type suggest perineural cell lineage.[7]

Atypical neurothekeomas have been described as resembling either benign or malignant melanocytic growths such as malignant melanoma, Spitz nevus, and cellular blue nevus. In contrast to melanomas that are S-100 positive, cellular neurothekeomas characteristically do not stain with antibody to S-100 protein.[8]

Clinically, neurothekeomas are slow-growing asymptomatic lesions. They are commonly dermal, but mucosa and submucosal lesions have been described.[4] It is a tumor of the adolescent and the young adult, with female predilection, extremely rare in infancy. In a literature review of a constellation of 292 cases of neurothekeoma, the age of presentation ranged from 15 months to 84 years.[9] Our case is the first ever presentation in an infant (less than 12 months). The most common location was the upper extremity, followed by the head and the neck, the trunk, and the lower extremities.[1,9] Treatment of neurothekeoma is complete excision. Recurrence of the tumor was thought to be secondary to incomplete removal of the original lesion.[1]

## Conclusion

Neurothekeoma is extremely rare in infancy. Myxoid neurothekeoma may present as dermal lesion in the finger. Reporting such a case will increase awareness about this disease, where it should be included in the differential diagnosis of dermal lesion in infancy and young children. Histopathology with immunostaining helps in diagnosing, classifying (between myxoid, cellular or mixed varieties) and differentiating (between melanocytic, Spitz nevus, neurofibroma etc) among other soft tissue lesions. Complete surgical excision is curative for these lesions.
